# Cardiovascular Disease and COVID-19: Is There a Place for Mid-Regional Pro-Adrenomedullin? Preliminary Data from a Clinical Cohort

**DOI:** 10.3390/ijms262010081

**Published:** 2025-10-16

**Authors:** Paulina Pietraszko, Marcin Żórawski, Emilia Bielecka-Richter, Małgorzata Gryciuk, Kacper Mil, Aleksandra Zbroch, Edyta Zbroch

**Affiliations:** 1 Department of Internal Medicine and Hypertension, Medical University of Bialystok, Zurawia 14, 15-540 Bialystok, Poland; pietraszko270@gmail.com (P.P.); emilka.bielecka96@gmail.com (E.B.-R.); mgryciuk@gmail.com (M.G.); mil.kacper@wp.pl (K.M.); olaz@onet.pl (A.Z.); 2 Clinical Department of Cardiology, Lipidology and Internal Medicine with Cardiac Intensive Care Unit, Zurawia 14, 15-540 Bialystok, Poland; marcin.zorawski@umb.edu.pl

**Keywords:** COVID-19, pro-adrenomedullin, cardiovascular complications, biomarkers, preliminary study

## Abstract

Mid-regional pro-adrenomedullin (MR-proADM) has emerged as a promising biomarker reflecting endothelial dysfunction and systemic inflammation. Its prognostic role in cardiovascular complications, particularly in the context of COVID-19 infection, remains under investigation. This study aimed to evaluate MR-proADM concentrations in patients with and without cardiovascular disease and COVID-19 and to assess its association with cardiac complications and biomarkers of myocardial injury. A total of 157 patients (mean age: 72.3 years; 66 men) hospitalized in a tertiary referral center were enrolled. The study population consisted of three groups: patients with cardiovascular disease and COVID-19 (*n* = 64), patients with cardiovascular disease but no COVID-19 (*n* = 74), and a control group without cardiovascular disease or COVID-19 (*n* = 17). Plasma MR-proADM levels were measured, and their relationship with cardiovascular complications (chronic heart failure and myocardial infarction) and standard cardiac biomarkers (troponin T, troponin I, and proBNP) was analyzed. The mean MR-proADM concentration in the overall cohort was 91.48 ± 71.96 pmol/L (median: 77.95; range: 5.81–429.20). Distributions of MR-proADM, troponin T (M = 143.12 ± 733.93 ng/L), troponin I (M = 143.37 ± 749.85 ng/L), and proBNP (M = 2080.29 ± 3632.03 pg/mL) deviated significantly from normality (Shapiro–Wilk, all *p* < 0.001). No significant differences in MR-proADM concentrations were observed between patients with COVID-19 vs. without (93.78 ± 56.94 vs. 92.08 ± 82.82 pmol/L, *p* = 0.842) and between active infection vs. past COVID-19 (93.78 ± 56.94 vs. 85.98 ± 50.39 pmol/L, *p* = 0.869). A trend toward higher MR-proADM concentrations was observed in patients with cardiovascular disease compared to those without, with mean levels of 93.56 ± 74.90 vs. 74.33 ± 37.43 pmol/L, respectively. The frequency of chronic heart failure (55.0% vs. 54.5%, *p* = 1.000) and myocardial infarction (11.3% vs. 20.8%, *p* = 0.128) did not differ between patients with low vs. high MR-proADM (cut-off: median). Logistic regression confirmed that MR-proADM did not significantly predict either chronic heart failure (Nagelkerke R^2^ = 0.002, *p* = 0.660) or myocardial infarction (Nagelkerke R^2^ = 0.006, *p* = 0.465). Correlation analysis showed no significant associations between MR-proADM and proBNP (ρ = 0.09, *p* = 0.323), troponin T (ρ = 0.22, *p* = 0.065), or troponin I (ρ = 0.16, *p* = 0.088). MR-proADM levels did not differ significantly between patients stratified by COVID-19 infection or cardiovascular disease and were not predictive of heart failure or myocardial infarction. Moreover, no correlations were found with standard cardiac biomarkers. These results suggest that, in this cohort, MR-proADM did not provide additional prognostic information for cardiovascular complications.

## 1. Introduction

The outbreak of COVID-19 has impacted populations across the globe [1], and it soon became evident that certain groups of patients are particularly vulnerable to unfavorable outcomes. One of the major challenges in the management of this infection is the significantly worse prognosis observed in individuals with predisposing factors. Consequently, numerous strategies have been undertaken to reduce the burden of the disease and limit its adverse health consequences [2]. Several demographic and clinical variables—including advanced age, obesity, male sex, ethnic background, and pre-existing comorbidities, especially cardiovascular disorders such as arterial hypertension and chronic heart failure—have consistently been linked with a higher risk of severe disease progression and mortality [3].

In addition, COVID-19 infection itself frequently triggers cardiovascular involvement. Reported complications include thromboembolic events, myocarditis, arrhythmias, acute myocardial injury, and decompensation of underlying heart disease, all of which contribute substantially to the morbidity and mortality associated with SARS-CoV-2 infection [4,5].

**Figure 1:**
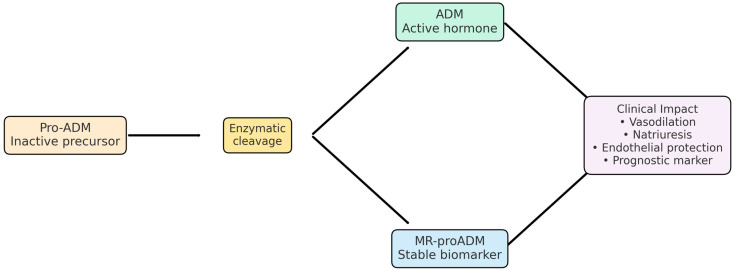
Adrenomedullin (ADM)/MR-proADM pathway and clinical relevance. Pro-ADM undergoes enzymatic cleavage, yielding biologically active adrenomedullin and the stable fragment MR-proADM, which serves as a biomarker. ADM exerts vasodilatory, natriuretic, and endothelial-protective effects and has been proposed as a prognostic marker in cardiovascular and infectious diseases [10,11,12].

Within this context, mid-regional pro-adrenomedullin (MR-proADM) has emerged as a promising biomarker. MR-proADM is a stable fragment of the precursor molecule of adrenomedullin (ADM), a peptide originally discovered in human pheochromocytoma tissue [6]. ADM exerts potent vasodilatory, natriuretic, and anti-inflammatory effects and plays a pivotal role in maintaining endothelial barrier function and vascular homeostasis. The biological activity of ADM is mediated through the calcitonin receptor-like receptor (CLR), which requires interaction with receptor activity-modifying proteins (RAMP2 or RAMP3) to form functional adrenomedullin receptors (AM1 and AM2). Through these receptor complexes, ADM influences vascular smooth muscle tone, reduces oxidative stress, and protects against endothelial apoptosis [7,8,9] (Figure 1).

Circulating levels of MR-proADM are elevated in a variety of pathological conditions. Increased concentrations have been described in cardiovascular diseases such as hypertension, acute and chronic heart failure, and myocardial infarction [13,14,15]. MR-proADM exerts its biological effects on multiple cellular targets within the cardiovascular system, including vascular smooth muscle cells, endothelial cells, and cardiomyocytes. By inducing vasodilation in the peripheral vasculature, ADM decreases systemic vascular resistance, which in turn contributes to the lowering of arterial blood pressure [16]. In addition, ADM has been shown to improve coronary perfusion, facilitating greater blood flow within the coronary circulation [17]. Beyond its hemodynamic properties, ADM demonstrates cardioprotective actions, such as limiting myocardial damage and supporting recovery of cardiac function during ischemic episodes [13].

Beyond cardiovascular pathology, MR-proADM has also been recognized as a biomarker of infectious diseases, where it reflects the systemic response to inflammation and circulatory stress. Its concentrations rise significantly in the setting of severe infections, particularly in sepsis, where endothelial injury and microcirculatory dysfunction play a central role in pathogenesis. Elevated MR-proADM levels in septic patients have been linked to increased vascular permeability, impaired tissue oxygenation, and progression toward multiple organ failure. Several clinical studies have consistently demonstrated that higher MR-proADM values are associated with poor outcomes, including prolonged intensive care unit (ICU) stay, need for vasopressor support, and higher mortality rates. These findings highlight the close relationship between MR-proADM and the integrity of the endothelium as well as its value as a prognostic indicator in systemic infections [18,19,20,21,22,23,24,25,26].

Recent evidence suggests that a similar pattern is observed in COVID-19. Patients infected with SARS-CoV-2 frequently exhibit elevated MR-proADM concentrations, which are thought to mirror the degree of endothelial injury, widespread inflammation, and multiorgan involvement characteristic of the disease. In severe cases, MR-proADM has been shown to correlate with clinical deterioration, respiratory failure, and adverse cardiovascular events, supporting its potential role as a biomarker of disease severity. Nonetheless, despite accumulating data, the precise contribution of MR-proADM in different phases of COVID-19—during acute infection and in the post-COVID state—remains to be fully clarified. In particular, its usefulness in predicting or monitoring cardiovascular complications is still under investigation and warrants further study [27,28].

Therefore, the aim of this preliminary study was to investigate the overall significance of MR-proADM in patients with active COVID-19 and in the post-COVID phase, specifically in the context of cardiovascular complications.

## 2. Results

In the first step of the analysis, the distribution of quantitative variables was examined. Descriptive statistics were calculated together with the Shapiro–Wilk test for normality. The results are presented in Table 1. For all variables under study, the Shapiro–Wilk test indicated statistically significant deviations from normality. In addition, skewness values greater than two were observed, suggesting substantial right-sided asymmetry. Based on these findings, further analyses were performed using non-parametric tests.

### 2.1. Comparison of MR-proADM Levels Across Study Groups

Subsequently, the study groups were compared in terms of MR-proADM concentrations. The Mann–Whitney U test was used to evaluate differences between the study and control cohorts, between patients with and without cardiovascular disease, and between individuals with active versus past COVID-19 infection. The results are summarized in Table 2.

No statistically significant differences were observed between the compared groups. This indicates that MR-proADM concentrations did not differ between patients with and without COVID-19, between those with or without cardiovascular disease, or between patients with active versus past infection.

### 2.2. Cardiovascular Complications and MR-proADM Levels

Next, the analysis focused on the potential association between MR-proADM concentrations and the occurrence of cardiovascular complications, specifically chronic heart failure and myocardial infarction. Patients were divided into subgroups according to low and high MR-proADM levels, with the median value serving as the cut-off point. The chi-square test of independence was used to compare the frequency of complications between these groups. The results are presented in Table 3.

No statistically significant differences were found in the frequency of chronic heart failure or myocardial infarction between patients with low and high MR-proADM concentrations. This suggests that MR-proADM levels were not associated with the risk of these complications. To further explore this relationship, logistic regression models were constructed to assess whether MR-proADM levels predicted the occurrence of chronic heart failure or myocardial infarction. The results are presented in Table 4.

The models were not well fitted to the data, explaining only a negligible proportion of variance in the outcomes. The logistic regression models demonstrated very low explanatory power, with Nagelkerke R^2^ values close to zero (0.002 for chronic heart failure and 0.006 for myocardial infarction). MR-proADM concentration was not identified as a statistically significant predictor of either chronic heart failure or myocardial infarction. The logistic regression models were poorly fitted to the data, explaining only a negligible proportion of variance in the outcomes. Nagelkerke R^2^ values were close to zero (0.002 for chronic heart failure and 0.006 for myocardial infarction), indicating very low explanatory power. Moreover, MR-proADM concentration was not identified as a statistically significant predictor of either chronic heart failure or myocardial infarction

### 2.3. Correlation Between MR-proADM and Cardiac Biomarkers

Finally, the relationship between MR-proADM concentrations and markers of cardiac injury was analyzed. Spearman’s rho correlation coefficients were calculated for MR-proADM, proBNP, troponin T, and troponin I. The results are presented in Table 5.

No statistically significant associations were found between MR-proADM and the studied cardiac biomarkers. This indicates that changes in MR-proADM concentrations were not correlated with changes in proBNP, troponin T, or troponin I.

### 2.4. MR-proADM Concentrations According to COVID-19 Severity

**Figure 2:**
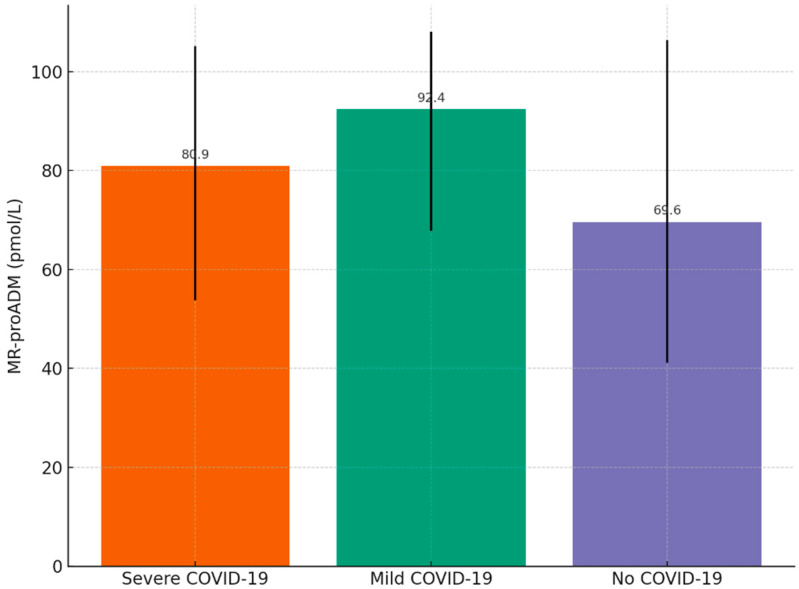
Median MR-proADM concentrations with interquartile ranges (IQRs) in patients with severe COVID-19, mild COVID-19, and no history of COVID-19 infection.

Patients with documented cardiovascular disease and COVID-19 infection were further subdivided according to disease severity. A severe course was defined as hospitalization for COVID-19 requiring guideline-based therapy, including systemic corticosteroids and/or antiviral treatment with remdesivir. A mild course was defined as laboratory-confirmed SARS-CoV-2 infection not requiring pharmacological therapy. Patients without a history of COVID-19 served as the reference group. Median MR-proADM concentrations showed a non-significant tendency toward higher values in COVID-19 groups compared with patients without a history of infection (Table 6). Patients with a mild course of COVID-19 demonstrated the highest median MR-proADM concentration (92.4 pmol/L, IQR 67.8–108.0), followed by those with a severe course (80.9 pmol/L, IQR 53.8–105.1), while patients without COVID-19 had lower median values (69.6 pmol/L, IQR 41.2–106.4). Kruskal–Wallis testing indicated no statistically significant differences among the groups. The graphical representation (Figure 2) illustrates the trend toward higher MR-proADM levels in patients with COVID-19 compared with non-COVID individuals.

## 3. Discussion

The present study evaluated the role of mid-regional pro-adrenomedullin (MR-proADM) in predicting cardiovascular complications in patients with and without COVID-19 infection. Although previous investigations have suggested that MR-proADM may serve as a reliable biomarker of endothelial dysfunction, inflammation, and adverse cardiovascular outcomes, our results did not demonstrate significant associations between MR-proADM concentrations and the occurrence of myocardial infarction, heart failure, or arrhythmias. Furthermore, no statistically significant correlations were observed between MR-proADM and conventional cardiac biomarkers such as troponins or proBNP. These findings warrant careful consideration in the broader context of existing literature.

Adrenomedullin is a vasoactive peptide with pleiotropic effects on the cardiovascular system, including vasodilation, regulation of vascular permeability, and protection against oxidative stress and inflammation [29]. MR-proADM, a stable fragment derived from the precursor molecule, is considered a surrogate biomarker that reflects ADM activity and has been extensively studied in cardiovascular and infectious diseases [20,30]. Several studies have demonstrated that elevated MR-proADM levels are strongly associated with disease severity, organ dysfunction, and mortality in patients with sepsis and pneumonia [31,32]. In the context of COVID-19, MR-proADM has attracted attention as a prognostic biomarker due to its potential to reflect endothelial injury and systemic inflammation, which are hallmarks of severe SARS-CoV-2 infection [33,34,35].

The cardiovascular implications of COVID-19 have been widely recognized, with myocardial injury, arrhythmias, thromboembolic events, and heart failure occurring in a substantial proportion of hospitalized patients [36,37]. MR-proADM has been proposed as a promising tool to stratify risk in this population. Indeed, a number of studies have reported that higher MR-proADM levels are predictive of in-hospital mortality and adverse cardiovascular events among COVID-19 patients [38,39,40]. For example, Astapovskii et al. (2022) demonstrated that MR-proADM exhibited higher prognostic value than routine inflammatory markers—including leukocyte and neutrophil counts, C-reactive protein (CRP), procalcitonin (PCT)—as well as the National Early Warning Score (NEWS), for predicting outcomes in COVID-19 patients [41]. Similarly, García de Guadiana-Romualdo et al. observed that MR-proADM levels > 1.8 nmol/L were strongly associated with both 28-day mortality and the need for intensive care unit (ICU) admission [39]. These findings highlight the clinical utility of MR-proADM as a marker of endothelial dysfunction and disease progression in COVID-19.

In contrast, our study did not identify significant differences in MR-proADM concentrations between patients with active COVID-19, those with prior infection, and controls without COVID-19. This discrepancy may be attributed to several factors. First, our cohort consisted of patients with pre-existing cardiovascular disease, which itself may elevate MR-proADM levels irrespective of acute infection status [42,43,44]. It is plausible that the underlying cardiovascular pathology masked potential differences attributable to COVID-19. Second, the relatively small sample size of our subgroups, particularly in the post-COVID-19 cohort, may have limited the statistical power to detect differences. Third, heterogeneity in disease severity within the COVID-19 group may have diluted potential associations, as the biomarker appears to be most informative in critically ill patients requiring ICU-level care [45].

Furthermore, our data revealed a non-significant trend toward higher MR-proADM medians in both mild and severe COVID-19 subgroups compared to patients without infection. Despite not reaching statistical significance—likely reflecting sample size and clinical heterogeneity—this trend aligns directionally with larger studies suggesting MR-proADM elevation may mirror underlying endothelial stress even post-acute SARS-CoV-2 infection [46,47].

Beyond COVID-19, MR-proADM has been extensively investigated in the context of chronic heart failure (CHF). Elevated plasma MR-proADM concentrations have consistently been associated with increased mortality, adverse remodeling, and risk of hospitalization in CHF cohorts [47,48]. Maisel et al. reported that MR-proADM was an independent predictor of 90-day mortality in patients presenting with acute decompensated heart failure, outperforming natriuretic peptides in prognostic discrimination [31]. Similarly, in the study by Johnsson et al. [49], higher levels of bio-adrenomedullin (bio-ADM) at hospital admission were linked to the later development of acute respiratory distress syndrome (ARDS), regardless of whether patients already had sepsis or organ dysfunction, as measured by the Sequential Organ Failure Assessment (SOFA) score. Interestingly, both very low concentrations (<38 pg/L) and very high concentrations (>90 pg/L) of bio-ADM were strong independent predictors of mortality, even when compared with the Simplified Acute Physiology Score (SAPS-3). The researchers also observed that patients with indirect causes of lung injury, such as sepsis, showed higher bio-ADM levels than those with direct causes of lung injury, such as pneumonia or aspiration. Moreover, bio-ADM levels increased together with the severity of ARDS, suggesting that this biomarker could be valuable for monitoring disease progression over time. The peptide’s ability to integrate signals of hemodynamic stress, endothelial dysfunction, and systemic inflammation likely explains its strong prognostic performance in this setting.

Despite these promising data, our results did not confirm a significant predictive role of MR-proADM for chronic heart failure in the study population. The logistic regression models revealed minimal explanatory power, with Nagelkerke’s R^2^ below 1%. Several explanations may account for this lack of association. One possibility is that MR-proADM may primarily reflect acute hemodynamic and inflammatory stress rather than chronic stable disease states. Indeed, prior studies demonstrating prognostic value were predominantly performed in acutely decompensated patients [48,49,50]. Our population, by contrast, included many clinically stable individuals with chronic cardiovascular disease, in whom biomarker fluctuations may have been less pronounced. Our findings are consistent with those reported in the study by Fraty et al. [51], which investigated the prognostic value of MR-proADM in a large cohort of patients with type 2 diabetes. In that prospective analysis, elevated MR-proADM levels independently predicted the occurrence of congestive heart failure (CHF). However, the biomarker did not provide significant additional predictive information beyond N-terminal pro-B-type natriuretic peptide (NT-proBNP), which remained the stronger tool for risk assessment.

The analysis of myocardial infarction yielded similar results. While MR-proADM has been linked to worse outcomes following acute coronary syndromes in some studies [52,53], including higher rates of reinfarction and mortality, we found no evidence of predictive utility. This again may be explained by differences in study design and patient populations. In acute coronary syndrome cohorts, MR-proADM likely reflects the extent of ischemia–reperfusion injury, systemic inflammation, and microvascular dysfunction, which contribute to adverse outcomes [54]. In our sample, however, myocardial infarction was a historical event in many patients, and biomarker measurements were performed in a stable setting, reducing the ability to capture these acute pathophysiological processes.

Interestingly, no significant correlations were observed between MR-proADM and conventional biomarkers such as troponins or proBNP. This contrasts with previous evidence suggesting modest associations between MR-proADM and natriuretic peptides [55]. For instance, Ara-Somohano et al. [56] evaluated eight different biomarkers in patients admitted with acute severe dyspnea and found that MR-proADM demonstrated the highest accuracy in predicting 28-day outcomes, outperforming the other tested biomarkers. In line with these findings, Stokes et al. [57] demonstrated that MR-proADM outperformed BNP and NT-proBNP in identifying patients with acute decompensated heart failure who were at the highest risk of 90-day mortality, highlighting its stronger prognostic value in the setting of acute dyspnea. The absence of such associations in our study may again be related to the relative clinical stability of our patients and the timing of biomarker measurement outside of acute decompensation.

Taken together, our findings suggest that MR-proADM may have limited utility as a prognostic biomarker in stable patients with cardiovascular disease, regardless of prior or current COVID-19 infection. Instead, its clinical role may be more pronounced in acutely ill populations, particularly those with sepsis, pneumonia, or acute decompensated heart failure, where endothelial dysfunction and systemic inflammation are dominant drivers of pathology [39,48]. The observed discrepancies also underscore the importance of contextualizing biomarker findings according to disease stage, severity, and timing of measurement.

An additional finding of our analysis was the lack of significant differences in MR-proADM concentrations between patients with or without underlying cardiovascular disease. This is somewhat surprising, given prior reports linking MR-proADM to atherosclerotic burden, endothelial dysfunction, and vascular remodeling [58,59,60]. For instance, Roos et al. [58] in their study demonstrated that higher MR-proADM levels were associated with increased carotid intima-media thickness and incident cardiovascular events in a population-based cohort. Likewise, a large analysis by Melander et al. [59] showed that MR-proADM independently predicted future cardiovascular morbidity and mortality in initially healthy individuals. These data highlight the potential of MR-proADM as a biomarker of subclinical vascular pathology. However, in our study, the lack of association with established cardiovascular disease may be due to the presence of multiple overlapping risk factors, medication effects (e.g., statins and ACE inhibitors), and the chronicity of disease, which could obscure biomarker differences.

It is important to emphasize that MR-proADM is not a disease-specific biomarker but rather a global indicator of endothelial stress, inflammation, and vascular dysfunction. Consequently, its predictive performance may vary substantially across clinical contexts. In sepsis and pneumonia, MR-proADM has consistently emerged as one of the strongest predictors of mortality, outperforming classical inflammatory markers such as CRP and procalcitonin [39,41]. In cardiovascular medicine, its utility appears to be maximized in acute and unstable states such as acute heart failure, myocardial infarction, and cardiogenic shock [43,44,48]. By contrast, in stable outpatient populations, its discriminatory value may be limited, as seen in our analysis.

Another aspect worth discussing is the absence of correlation between MR-proADM and troponin levels in our cohort. Troponins are highly specific markers of cardiomyocyte injury and necrosis, whereas MR-proADM reflects endothelial dysfunction and systemic stress. The lack of correlation suggests that these biomarkers provide complementary rather than overlapping information. Indeed, prior studies have shown that combining MR-proADM with troponins or natriuretic peptides can enhance risk stratification [61,62]. For example, Maisel et al. [31] demonstrated that patients with concordantly elevated MR-proADM and BNP had the highest risk of short-term mortality, while discordant biomarker patterns carried intermediate risk. Thus, even if MR-proADM alone was not predictive in our cohort, it may still have potential utility when integrated into multimarker strategies.

The negative findings of our logistic regression models should not be interpreted as definitive evidence against the prognostic value of MR-proADM in cardiovascular disease. Rather, they highlight the complexity of biomarker research. Factors such as sample size, patient heterogeneity, disease stage, comorbidities, and timing of measurement can profoundly influence observed associations. In addition, statistical models may underestimate the contribution of biomarkers when outcomes are influenced by numerous interacting clinical factors. Future studies with larger sample sizes, longitudinal follow-up, and stratification by disease severity are needed to clarify the role of MR-proADM in predicting cardiovascular complications in both COVID-19 and non-COVID-19 populations.

Our correlation analysis between MR-proADM and proBNP was also non-significant, which contrasts with earlier work. Natriuretic peptides reflect myocardial wall stress and are well-established prognostic markers in heart failure [63]. Several studies have reported weak-to-moderate correlations between MR-proADM and NT-proBNP, suggesting that these biomarkers capture partially overlapping but distinct aspects of pathophysiology [64,65]. The absence of correlation in our data may again reflect the relative clinical stability of our patients and the heterogeneity of underlying conditions.

In the context of COVID-19, the role of MR-proADM remains an area of intense investigation. Several multicenter studies have suggested that MR-proADM could outperform conventional inflammatory markers in identifying patients at risk of severe outcomes [33,39,40]. For instance, in the study by Popov et al. [40], conducted in patients hospitalized in the intensive care unit, MR-proADM showed the highest predictive value for mortality when compared with the NEWS score, peripheral oxygen saturation (SpO_2_), procalcitonin (PCT), and lymphocyte count. These findings underscore the biomarker’s potential to reflect the systemic endothelial dysfunction that characterizes severe SARS-CoV-2 infection. The discrepancy with our results likely reflects differences in disease severity, as most of our patients were not critically ill at the time of measurement.

### Limitations

One important limitation of our study is the cross-sectional design, which precludes assessment of dynamic biomarker changes over time. Prior studies suggest that serial MR-proADM measurements may provide stronger prognostic information than single baseline values [66,67]. Rising MR-proADM levels during hospitalization have been associated with worsening outcomes in both sepsis and heart failure, whereas stable or declining levels are linked to improved survival [67]. Future research should therefore incorporate longitudinal biomarker monitoring to better capture the dynamic interplay between endothelial dysfunction, inflammation, and cardiovascular events. Furthermore, medication use (e.g., ACE inhibitors, ARBs, and statins) may have influenced MR-proADM concentrations by modulating endothelial and inflammatory pathways, though these effects were not systematically controlled for in our analysis.

A further limitation is the relatively small sample size of some subgroups, particularly the post-COVID-19 cohort, which may have reduced the statistical power to detect subtle differences in MR-proADM concentrations. Consequently, the absence of statistically significant differences in the primary outcome across the three groups should be interpreted with caution, as it may reflect limited statistical power rather than a true lack of association. Another limitation worth mentioning is the single-center design, which may reduce the generalizability of the findings. Larger, multicenter, and prospective studies are required to confirm these preliminary results and establish the potential role of MR-proADM in cardiovascular risk assessment.

It should also be acknowledged that, although MR-proADM was evaluated in comparison with established cardiovascular biomarkers such as troponins and proBNP, other inflammatory markers, including CRP, were not consistently available and thus could not be analyzed. This may have limited the scope of biomarker validation. Additionally, the study population consisted of patients hospitalized in the Department of Internal Medicine, which by design excluded critically ill individuals requiring intensive care. This likely contributes to the divergence between our findings and those reported in ICU cohorts, where MR-proADM has demonstrated strong prognostic utility. Therefore, our results should be interpreted within the context of a general internal medicine population.

Another limitation is the inability to perform more detailed subgroup analyses stratifying patients by both COVID-19 severity (severe, mild, and no infection) and the presence of cardiovascular complications. Although such an approach could provide further insight into the relationship between MR-proADM and cardiovascular outcomes, the relatively small number of patients in several subgroups precluded meaningful statistical evaluation. Future studies with larger cohorts—including critically ill patients—should address this issue to better clarify the observed trends.

Despite the above limitations, our study contributes to the growing body of literature examining the prognostic role of MR-proADM in cardiovascular and infectious diseases. The absence of strong associations in our cohort suggests that MR-proADM may not be universally applicable as a prognostic tool in all patient populations. Rather, its utility may be greatest in acute, unstable conditions characterized by heightened endothelial dysfunction and inflammatory activation. This interpretation aligns with the broader biomarker literature, which increasingly emphasizes the context-specific nature of prognostic indicators [68].

## 4. Materials and Methods

The study cohort consisted of patients hospitalized in the Department of Internal Medicine and Hypertension, Medical University of Bialystok, Poland. Patient recruitment was carried out between 24 June 2022, and 19 December 2023. The inclusion criteria comprised individuals with a documented history of cardiovascular disease and a confirmed diagnosis or exclusion of COVID-19 infection based on polymerase chain reaction or antigen testing and verified through medical records. The total study population was divided into three groups. The first group included patients with cardiovascular disease and COVID-19 infection, either during the acute phase of illness or in the post-infectious period. The second group included patients with cardiovascular disease without any evidence of prior COVID-19 infection. The third group served as a control population and consisted of individuals without a history of cardiovascular disease and without documented COVID-19 infection. Eligibility was confirmed through a review of hospital medical records and cross-checked with the Polish National COVID-19 Patient Registry, a dedicated database established during the pandemic to monitor infected individuals. Controls were recruited among patients admitted to the Department of Internal Medicine for non-cardiovascular conditions, such as gastrointestinal or metabolic disorders. All participants were identified through available hospital documentation and confirmed by review of clinical history. Written informed consent was obtained from all participants prior to study enrollment. The study protocol was reviewed and approved by the Bioethics Committee of the Medical University of Bialystok (protocol code APK.002.41.7.2020), in accordance with the principles of the Declaration of Helsinki. Characteristics of the study population are presented in Table 7.

The study groups were further evaluated for demographic and anthropometric characteristics, including sex, age, body weight, height, and body mass index. Blood pressure values were collected from medical records. Detailed clinical data were extracted to assess cardiovascular risk factors and complications. Cardiovascular complications were defined as acute myocardial infarction, arrhythmias, or heart failure and were determined based on echocardiographic findings, electrocardiographic recordings, and biomarker measurements. The distribution of cardiovascular complications across study groups is summarized in Table 8.

### 4.1. Laboratory Examinations

Venous blood samples were collected from all participants in the morning, together with routine laboratory tests, into S-Monovette tubes (SARSTEDT, Nümbrecht, Germany). Samples were centrifuged immediately after collection—EDTA plasma, or, after approximately 60 min following complete clot formation, serum—at 1500× *g* for 15 min. Plasma and serum were then promptly aliquoted and stored at −80 °C until analysis. Plasma concentrations of MR-proADM were measured using a standardized commercial Human MR-proADM ELISA Kit (MBS2600007, MyBioSource, San Diego, CA, USA), following the manufacturer’s protocol. Serum concentrations of proBNP, troponin T, and troponin I were determined using immunoassays on the Abbott Alinity Analyzer (Abbott Laboratories, Abbott Park, IL, USA. All laboratory analyses were performed in the Central Laboratory of the Medical University of Bialystok.

### 4.2. Statistical Analysis

Statistical analyses were performed using dedicated statistical software. The normality of continuous variables was verified using the Shapiro–Wilk test. Variables with non-normal distribution were analyzed with the Mann–Whitney U test, while categorical variables were compared using the chi-square test of independence. Correlations between quantitative parameters not following a normal distribution were examined using the Spearman rank correlation coefficient. Logistic regression models were applied to determine predictors of cardiovascular complications. To assess interactions between factors, a two-way analysis of variance was performed. A *p* value below the level of statistical significance was considered indicative of a meaningful association.

In the next phase of the study, the analyses will be expanded to explore the association between MR-proADM levels and renal function, including both documented chronic kidney disease (CKD) and acute kidney injury (AKI). Additionally, the study cohort will be broadened to include patients diagnosed with kidney disease at various stages.

## 5. Conclusions

In conclusion, our findings suggest that MR-proADM has limited prognostic value for chronic cardiovascular complications in stable patients, regardless of COVID-19 status. However, existing evidence from larger and acutely ill cohorts supports its role as a powerful predictor of short-term mortality and adverse events in sepsis, pneumonia, acute heart failure, and severe COVID-19. Future studies should aim to clarify the contexts in which MR-proADM measurement is most informative, explore its integration with other biomarkers, and evaluate its potential role in guiding therapeutic decision-making.

## Figures and Tables

**Table 1:** Descriptive statistics and Shapiro–Wilk test for normality.

Variable	Mean	Median	SD	Skewness	Kurtosis	Min	Max	W, *p*-Value
MR-proADM	91.48	77.95	71.96	2.16	5.90	5.81	429.20	0.80, <0.001
proBNP	2080.29	782.90	3632.03	3.52	14.65	10.70	23,498.00	0.63, <0.001
Troponin T	143.12	20.00	733.93	8.14	68.25	4.00	6251.00	0.40, <0.001
Troponin I	143.37	10.00	749.85	7.10	52.71	5.00	6466.00	0.18, <0.001

**Table 2:** Comparison of MR-proADM concentrations across study groups.

Group	MR-proADM			*Z*	*p*	η^2^
	Mean Rank	*M*	*SD*			
Control group (*n* = 93)	74.70	91.66	82.09	−1.43	0.153	0.01
Study Group (*n* = 64)	85.25	91.22	54.60			
Group without cardiovascular disease (*n* = 17)	72.94	74.33	37.43	−0.58	0.561	<0.01
Group with cardiovascular disease (*n* = 140)	79.74	93.56	74.90			
Post COVID-19 group (*n* = 21)	31.95	85.98	50.39	−0.16	0.869	<0.01
Active COVID-19 group (*n* = 43)	32.77	93.78	56.94			

**Table 3:** Comparison of patients with low and high MR-proADM concentrations in terms of the prevalence of chronic heart failure and myocardial infarction.

		Low MR-proADM		High MR-proADM		χ^2^(1)	*p*	ϕ
		*n*	%	*n*	%			
Chronic Heart Failure	No	36	45.0%	35	45.5%	<0.01	1.000	<0.01
	Yes	44	55.0%	42	54.5%			
History of Myocardial Infraction	No	71	88.8%	61	79.2%	2.66	0.128	0.13
	Yes	9	11.3%	16	20.8%			

**Table 4:** Logistic regression models for MR-proADM as a predictor of complications.

Dependent Variable *	B	SE	Wald	*p*-Value	Exp(B)	95% CI LL	95% CI UL
Chronic heart failure	<0.01	<0.01	0.19	0.660	1.00	1.00	1.00
Constant	0.28	0.26	1.17	0.279	1.33		
Myocardial infarction	<0.01	<0.01	0.54	0.465	1.00	1.00	1.01
Constant	−1.86	0.35	28.13	<0.001	0.16		

* Model fit statistics: chronic heart failure: χ^2^(1) = 0.19, *p* = 0.660; Nagelkerke R^2^ = 0.002. Myocardial infarction: χ^2^(1) = 0.51, *p* = 0.477; Nagelkerke R^2^ = 0.006.

**Table 5:** Correlation between MR-proADM and cardiac biomarkers.

Variable	Spearman Rho	*p*-Value
proBNP	0.09	0.323
Troponin T	0.22	0.065
Troponin I	0.16	0.088

**Table 6:** Median MR-proADM concentrations with interquartile ranges (IQRs) in patients with severe COVID-19, mild COVID-19, and without COVID-19.

Group	*n*	Median (pmol/L)	IQR (Q1–Q3)
Severe COVID-19	36	80.9	53.8–105.1
Mild COVID-19	26	92.4	67.8–108.0
No COVID-19	93	69.6	41.2–106.4

**Table 7:** Characteristics of the study population.

Group	N	Males (*n*)	Mean Age, Years (±SD)
CVD + COVID	64	25	75.9 ± 10.1
CVD_noCOVID	76	32	74.3 ± 12.0
Healthy	17	9	50.3 ± 15.1
Total	157	66	72.3 ± 12.6

Values are expressed as mean ± standard deviation for continuous variables and as absolute numbers for categorical variables.

**Table 8:** Detailed cardiovascular complications in the study groups.

Complication	CVD + COVID (*n* = 64)	CVD Without COVID (*n* = 76)	Healthy Controls (*n* = 17)
Atrial fibrillation	6 (9.4%)	5 (6.6%)	0 (0.0%)
Carotid atherosclerosis	7 (10.9%)	27 (35.5%)	0 (0.0%)
Chronic heart failure	35 (54.7%)	51 (67.1%)	0 (0.0%)
Embolic events (during COVID)	4 (6.2%)	0 (0.0%)	0 (0.0%)
Embolic events (history)	6 (9.4%)	9 (11.8%)	0 (0.0%)
Impaired diastolic function	27 (42.2%)	35 (46.1%)	2 (11.8%)
Impaired systolic function	22 (34.4%)	31 (40.8%)	0 (0.0%)
Ischemic stroke	6 (9.4%)	3 (3.9%)	0 (0.0%)
Myocardial infarction	14 (21.9%)	11 (14.5%)	0 (0.0%)
Paroxysmal atrial fibrillation	8 (12.5%)	15 (19.7%)	0 (0.0%)
Permanent atrial fibrillation	15 (23.4%)	15 (19.7%)	0 (0.0%)
Persistent atrial fibrillation	6 (9.4%)	6 (7.9%)	0 (0.0%)
Supraventricular arrhythmias > 1%	4 (6.2%)	10 (13.2%)	0 (0.0%)
Ventricular arrhythmias > 1%	2 (3.1%)	7 (9.2%)	0 (0.0%)

## Data Availability

The data presented in this study are contained within the article. No additional database or publicly archived datasets were created or analyzed in this study.
